# Correction: Multi-Scale Effects of Nestling Diet on Breeding Performance in a Terrestrial Top Predator Inferred from Stable Isotope Analysis

**DOI:** 10.1371/journal.pone.0104472

**Published:** 2014-07-29

**Authors:** The PLOS ONE Staff

There is an error in the title for Figure S2. The complete, correct Figure S2 title is: “Relationship between the diet diversity (H’) and the prey consumption specificity (PS_i_) at the territory level (*n*  =  71)”. Please see the corrected Figure S2 here.

There are formatting errors in Table S2 and in Table S3. Please see the corrected Table S2 and Table S3 here.

## Supporting Information

Figure S2 Relationship between the diet diversity (H′) and the prey consumption specificity (PSi) at the territory level (n  =  71).
(DOC)

Table S2 Explanatory variables used in the GLMMs to assess their potential effect on Bonelli’s eagle productivity, classified either as spatiotemporal parameters, breeding pair parameters or diet parameters.
(DOC)

Table S3 Summary of model parameter estimates and standard error of parameter estimates for each model included in the GLMMs. Models are showed following the same order as in Table 3.
(DOC)
