# Supplementary material for: Correction: Multi-Scale Effects of Nestling Diet on Breeding Performance in a Terrestrial Top Predator Inferred from Stable Isotope Analysis
Source: PLoS One. 2014 Jul 29;9(7):e104472. doi: 10.1371/journal.pone.0104472 (PMC4114972; doi:10.1371/journal.pone.0104472)
Supplement: Table S2 — Explanatory variables used in the GLMMs to assess their potential effect on Bonelli’s eagle productivity, classified either as spatiotemporal parameters, breeding pair parameters or diet parameters. (DOC) [file pone.0104472.s002.doc]

**Table S2. Explanatory variables used in the GLMMs to assess their potential effect on Bonelli’s eagle productivity, classified either as spatiotemporal parameters, breeding pair parameters or diet parameters.**

| ***Spatiotemporal parameters*** (categorical factors) |
| --- |
| TERRITORY: territory code. |
| POPULATION: Catalonia, France or Andalusia. |
| YEAR: 2008-2011. |
| ***Breeding pair parameters*** (categorical factors)  AGE OF THE BREEDING PAIR: adult (i.e. both individuals with an adult plumage) or non-adult pair (i.e. at least one individual with a non-adult plumage). |
| MATE REPLACEMENT: presence or absence of replacement (based on individual plumage patterns) of at least one member of the breeding pair in two consecutive years. |
| ***Diet parameters*** (continuous variables) |
| RABBIT CONSUMPTION: mean percentage of European rabbit in the fledglings’ diet per territory estimated by SIAR. |
| PARTRIDGE CONSUMPTION: mean percentage of red-legged partridge in the fledglings’ diet per territory estimated by SIAR. |
| DIET DIVERSITY (H’): Shannon-Weaver diversity index using the mean consumption percentage of main prey categories in the fledglings’ diet per territory estimated by SIAR. |
| PREY CONSUMPTION SPECIFICITY (PS_i_): measure of diet overlap between a given territory and the population as a whole. The territorial diet represents the mean percentage of main prey categories in the fledglings’ diet estimated by SIAR. |
| ***Interaction of parameters*** |
| (H’ * PS_i_): interaction between H’ and PS_i_. |
| H’ + H’^2^: quadratic effect of H’. |
| PS_i_ + PS_i_^2^: quadratic effect of PS_i_. |
